# Amarilloviruses of Aquatic Animals

**DOI:** 10.3390/pathogens15020160

**Published:** 2026-02-02

**Authors:** Frederick Kibenge, Molly Kibenge, Daniela Vargas, Marcos Godoy

**Affiliations:** 1 Department of Pathology and Microbiology, Atlantic Veterinary College, University of Prince Edward Island, Charlottetown, PEI C1A 4P3, Canada; mkibenge@upei.ca; 2 Laboratorio de Biotecnología Aplicada, Facultad de Medicina Veterinaria, Universidad San Sebastián, Sede de la Patagonia, Lago Panguipulli 1390, Puerto Montt 5501842, Chile; danielaavargaszuniga@gmail.com (D.V.); or marcos.godoy@ciba.cl (M.G.); 3 Centro de Investigaciones Biológicas Aplicadas (CIBA), Puerto Montt 5480000, Chile

**Keywords:** *Flaviviridae*, *Pestiviridae*, *Hepaciviridae*, *Jingmenvirus*, flavi-like viruses, pesti-like virus, *Cyclopterus lumpus* virus (CLuV), salmon flavivirus (SFV), infectious precocity virus (IPV), Wenzhou shark flavivirus, fish amarilloviruses, crustacean amarilloviruses

## Abstract

The family *Flaviviridae* has been expanded to include the highly divergent flavi-like viruses into three new families, *Flaviviridae*, *Pestiviridae*, and *Hepaciviridae*, in the order *Amarillovirales*. Classical flavivirids are small, enveloped viruses with positive-sense ssRNA genomes lacking a 3′ poly(A) tail and ~9.0–13.0 kb in length, with a single open reading frame (ORF) encoding structural proteins at the N-terminus and nonstructural proteins at the C-terminus. Members infect a wide range of mammals, birds, and insects, and many are host-specific and pathogenic. Although the *RNA-directed RNA polymerase (RdRP)* gene sequences of the flavi-like viruses group phylogenetically with those of classical flavivirids, flavi-like viruses often encode larger polyproteins and possess substantially longer genomes of up to ~40 kb, and some have a 3′ poly(A) tail. Their host range extends across the whole animal kingdom and angiosperm plants. This review describes the reported flavi-like viruses of aquatic animals, providing a meaningful update on all three new families in *Amarillovirales* that have been discovered using metagenomics in fish, crustaceans, mollusks, and echinoderms. These amarilloviruses include pathogenic viruses of aquatic animals, such as *Cyclopterus lumpus* virus (CLuV) detected in moribund lumpfish, and infectious precocity virus (IPV) found in iron prawn syndrome (IPS)-affected farmed giant freshwater prawns.

## 1. Introduction

In this review, the highly divergent flavi-like viruses found in aquatic animals are collectively referred to as amarilloviruses, named after the order *Amarillovirales*, which contains the new families of flavivirids. Flavi-like viruses are defined as RNA viruses related to members of the original *Flaviviridae*, but with properties that diverge from those characteristic of flaviviruses such as having different genome lengths and configurations, and host range [1]. In 2025, the International Committee on Taxonomy of Viruses (ICTV) approved the reclassification the family *Flaviviridae* and its expansion through the incorporation of the large number of additional flavi-like viruses into three new families, *Flaviviridae* (with five genera: *Orthoflavivirus*, *Tamanavirus*, *Termitovirus*, *Guaicovirus*, and *Jingmenvirus*), *Pestiviridae* (with five genera: *Arachnivirus*, *Orthopestivirus*, *Boletivirus*, *Chrysopivirus* and *Koshovirus*) and *Hepaciviridae* (with two genera: *Orthohepacivirus* and *Pegivirus*), and an unnamed family (represented as Clade IV with one floating flavi-like virus, diatom colony-associated ssRNA virus (DCAV)), in the established order *Amarillovirales* [1,2]. Additionally, there are unnamed genera (designated Lineages) in the three families (five Lineages in *Flaviviridae*, two in *Pestiviridae*, and two in *Hepaciviridae*) [2].

Before this reclassification of flavivirids, the only family included in the order *Amarillovirales* was *Flaviviridae*, which is derived from the Latin flavus, “yellow,” referring to yellow fever virus. This virus causes yellow fever in humans, characterized by jaundice (yellowing of the skin and the whites of the eyes). The classical flavivirids (i.e., the original family *Flaviviridae*) are small, enveloped viruses with positive-sense, non-segmented, linear, single-stranded (ss)RNA genomes lacking a 3′ poly(A) tail [3] and approximately 9.0–13.0 kb in length, with a common genome organization. They all have a single 8000–10,700 base open reading frame (ORF), which is translated and cleaved into structural proteins (core and envelope proteins) located at the N-terminus and nonstructural proteins (protease, helicase, and RNA-directed RNA polymerase (RdRP)) at the C-terminus. They are homologous in their RdRP gene, superfamily 2 helicase (NS3), and serine protease domain sequences [4]. Viruses of different flavivirid genera (*Hepacivirus*, *Orthoflavivirus*, *Pegivirus*, and *Pestivirus* in the original family *Flaviviridae* [5]) differ, however, in their polyprotein translation strategy, which may be 5′-cap-dependent (orthoflaviviruses) or driven through an internal ribosomal entry site (IRES) (other flavivirids), and virion formation [2]. Their host range is primarily mammals and birds [6]. Many are host-specific and pathogenic, such as hepatitis C virus (HCV) in the genus *Hepacivirus*, a major human pathogen, bovine virus diarrhea virus (BVDV) and classical swine fever virus (CSFV) in the genus *Pestivirus*, which cause serious disease in cattle and pigs, respectively, members of the genus *Pegivirus* associated with persistent infections of a wide range of mammalian species but not associated with disease, and the genus *Orthoflavivirus*, which includes 53 species, most of which are arboviruses (arthropod-borne viruses; viruses biologically transmitted by blood-sucking insects and ticks—i.e., vertebrate viruses capable of reproducing in their arthropod vector), and many are important human and veterinary pathogens (e.g., yellow fever virus, YFV; dengue virus, DENV; Zika virus, ZIKV; Japanese encephalitis virus, JEV; West Nile virus, WNV) [7]. In contrast, the majority of flavi-like viruses have been discovered outside the primarily mammalian and vector host range of classified flavivirids, being distributed across the animal kingdom, from poriferans (sponges) [8], cnidarians (jellies) [9], mollusks (squid) [10], arthropods (insects) [11]; diplurans and scorpions [12]; crustaceans [9,10,13], nematodes [14], platyhelminths [15,16] to echinoderms (sea cucumbers) [17]), hemichordates (acorn worms) [9], cartilaginous and bony fish [9,18,19,20,21,22], amphibians (frogs) [9,23], reptiles [21], stramenopiles (diatoms and oomycotes) [24,25] and in angiosperm plants [26,27,28].

Evidently, the highly divergent and much wider range of flavi-like viruses has increased considerably. Although these viruses often possess quite different genome organizations, genome lengths, and host ranges, their RdRP gene sequences group phylogenetically with those of classical flavivirids [12], allowing for a genomics-based reclassification and expansion of the former family *Flaviviridae*. Given that the RdRP gene is the most evolutionarily conserved gene within virus groups [29], this reorganization of flavivirids is extremely robust. Thus, the flavi-like viruses have genome organizations distinct from those of classical flavivirids. They encode larger polyproteins and possess longer genomes (the so-called ‘large-genome flavi-like viruses flaviviruses (LGFs)’) [18,30,31], substantially longer than the classical flavivirids [26,28,32], the longest to date being Maximus pesti-like virus’ of about 40 kb, representing an instance of a flavi-like virus achieving a genome size comparable to that of the order *Nidovirales* [8], and some have a 3′ poly(A) tail [33]. Moreover, the new *Flaviviridae* family now includes three genera of viruses with non-segmented genomes (*Orthoflavivirus*, *Tamanavirus*, and *Termitovirus*) and two genera of viruses with multi-segmented genomes (*Guaicovirus* with five segments and *Jingmenvirus* with four segments [2]). In the old family *Flaviviridae*, the “segmented flavi-like viruses” with a segmented genome of four to five segments were classified by the ICTV as an unclassified sub-genus in the *Orthoflavivirus* genus under the putative genus name *Jingmenvirus* [12,34,35]. Jingmenviruses are found in ticks, nematodes, mosquitoes, humans, cattle, monkeys, bats, rodents, sheep, tortoises, crustaceans, plants, and fungi, and they are endemic in a wide range of environments worldwide [34,35]. Jingmen tick virus (JMTV), species *Jingmenvirus rhipicephali* [2], is a novel emerging tick-borne virus and is pathogenic to humans [35]. Infectious precocity virus (IPV), which is found in iron prawn syndrome (IPS)-affected farmed giant freshwater prawns *Macrobrachium rosenbergii*, was shown to belong to a proposed new genus *Crustaflavivirus* [13], which is phylogenetically between *Jingmenvirus* and *Orthoflavivirus* [13].

Aquatic viral diseases are recognized as one of the principal natural limiting factors for the global aquaculture sector, acting as a major constraint on the production, profitability, and sustainability of the industry worldwide [36]. The aquaculture sector is also highly vulnerable to climate change, with over 90% of global production facing substantial risks from environmental stressors [37,38], with the industry not only highly susceptible to the spread of established pathogens but also to the induction of emerging infectious diseases under rising water temperatures [39]. Advances in viral metagenomic next-generation sequencing (mNGS) [40,41] have rapidly expanded the discovery of new viruses in aquatic animals (e.g., crustaceans, mollusks, fish, and marine mammals) that challenge the current virus taxonomic scheme, leading to ongoing updates by various study groups of the ICTV [42,43]. For example, following advancements in metagenomics [44] and genomics [45], the decision of the ICTV to classify viruses based on their genome sequence alone, without the need for isolation and demonstration of infectivity [45,46], have enabled the classification of viruses from more diverse environments and infecting hosts that cannot be cultivated under laboratory conditions. This review describes the reported amarilloviruses of aquatic animals, in order to advance our understanding of the wide range of flaviviruses that occur in aquatic animals. These viruses group with the three new families in the order *Amarillovirales* and have been discovered in various marine hosts, including fish, crustaceans, mollusks, echinoderms, poriferans, and cnidarians using metagenomics [8,9,10,13,17,18,19,20,33,47,48]. Some of them cause disease in species that are important to the aquaculture industry [13,20,33].

Table 1 lists the highly divergent flavi-like viruses found in aquatic animals. These amarilloviruses in the new family *Flaviviridae* include Wenzhou shark flavivirus found in both a Pacific spadenose shark (*Scoliodon macrorhynchos*) [48] and a gazami crab (*Portunus trituberculatus*) [10], which group with the *Orthoflavivirus* genus but are not known to be transmitted by arthropods, and the pathogenic fish flavivirus *Cyclopterus lumpus* virus (CLuV) detected in moribund lumpfish (*Cyclopterus lumpus*) [20], which groups with the *Tamanavirus* genus. The fish viruses in the new family *Hepaciviridae* include Wēnlǐng shark virus (WLSV) (in the genus *Orthohepacivirus*) discovered in the graceful catshark (*Proscyllium habereri*) [18], Wēnlǐng moray eel hepacivirus (in Lineage IIIt [2]) in *Gymnothorax reticularis*, Xiàmén guitarfish hepacivirus in *Rhinobatos hynnicephalus*, Xiàmén sepia Stingray hepacivirus in *Urolophus aurantiacus*, Western African lungfish hepacivirus in *Protopterus annectens*, Guangxi houndshark hepacivirus in *Mustelus manazo*, and Nanhai dogfish shark hepacivirus in *Squalus brevirostris*, and Nanhai ghost shark hepacivirus 1 and 2 in *Chimaera* sp. [19,37]. The fish flaviviruses that group with the new family *Pestiviridae* include Xiàmén fanray pesti-like virus (XFPV) in Lineage IIn [2] detected in *Platyrhina* sp., Nanhai dogfish shark pesti-like virus in *Squalus brevirostris*, and Wenzhou pesti-like virus 1 and 2 discovered in *Sphyrna lewini* and *Proscyllium habereri*, respectively [19,48]. 

## 2. Virus Characteristics

**Figure 1:**
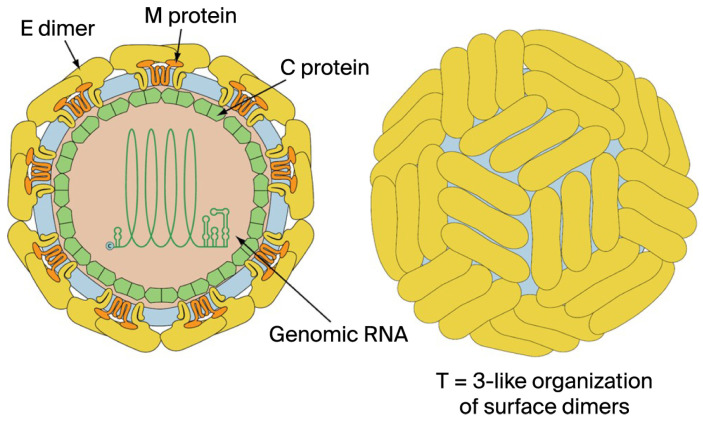
Schematic of the *Flaviviridae* virus particle. The left particle is a cross-section with the viral components labeled. Members of the old genera *Orthoflavivirus*, *Hepacivirus*, and *Pegivirus* have two E proteins (E dimers), whereas the genus *Pestivirus* has three (E trimers). The right particle shows the surface proteins arranged in an icosahedral-like symmetry. (Reproduced from [57]. Source: SwissBioPics. The images are licensed under a Creative Commons Attribution 4.0 International (CC BY 4.0) License https://creativecommons.org/licenses/by/4.0/).

**Figure 2:**
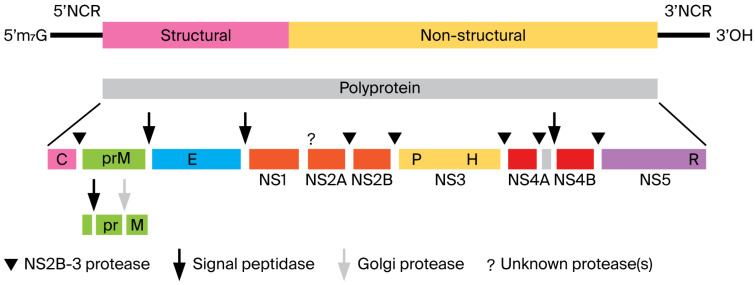
Genome organization and polyprotein processing of most classical flavivirids. Boxes below the genome indicate viral proteins generated by proteolytic processing. NCR, non-coding region. (Reproduced from [7], Figure 2, an open-access article distributed under the terms of the https://creativecommons.org/licenses/by/4.0/).

The virions of classical flavivirids (i.e., the original family *Flaviviridae*) are spherical, enveloped, and 37–60 nm in diameter (Figure 1), with a positive-sense ssRNA genome of about 9.5 kb (hepaciviruses) to 12.5 kb (pestiviruses), lacking a 3′ poly(A) tail [3]. Infectious precocity virus (IPV) particles are 40–60 nm in diameter with a genome of 12,630 nt in length [13]. Only the genomes of members of the genus *Orthoflavivirus* contain a 5′-methylated nucleotide cap (to allow translation). In contrast, others have a genome-linked protein (VPg) and an internal ribosomal entry site (IRES). All flavivirids encode a single large polyprotein, which is cleaved at conserved sites by either the viral serine protease (NS2B/NS3), a host-derived signalase, except for pr/M, which is cleaved after assembly by host-derived furin or a furin-like protease [54]. Thus, the flavivirid polyprotein is effectively processed into at least ten viral proteins: nonstructural (NS) proteins NS1, NS2A, NS2B, NS3, NS4A, NS4B, and NS5, and structural proteins designated C (core) and M (membrane-like), and glycoprotein (E) [13,55,56]. The polyprotein in almost all classical flavivirids is translated from a single ORF, with structural proteins located on the 5′ end (Figure 2) [7].

**Figure 3:**
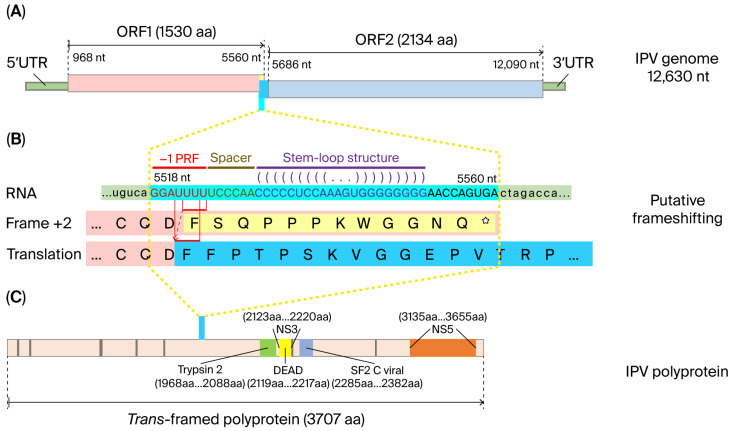
Production of a “transframe” polyprotein from two open reading frames (ORFs) utilizing programmed-1 ribosomal frameshifting (−1 PRF), using the IPV genome as an example. (**A**) The full-length genome of IPV with two predicted ORFs, 59 and 39 untranslated regions (39 UTRs), and potential translational changes caused by the putative frameshifting are indicated. The light-yellow block represents the frameshift-off region, taken over by the sky-blue block representing the frameshift-on region. The aqua column indicates the RNA sequence region, which is zoomed out in panel (**B**). (**B**) The putative frameshifting process. Nucleotide-level details of the aqua column of panel (**A**) are presented with the capital RNA sequence with aqua background, in which red letters indicate the potential 21 programmed ribosomal frameshifting (21 PRF), coffee letters indicate the 6-nt spacer, and dark magenta letters indicate the potential stem-loop structure. The red arrow with a dotted line indicates the putative frameshifting. The baby pink background indicates the last fragment of ORF1, in which the hollow letters with a light-yellow background indicate the frameshift-off amino acid sequence. The black letters with a sky-blue background indicate the frameshift-on amino acid sequence. (**C**) The predicted conserved domains in the polyprotein. (Reproduced from [13], Figure 3, an open-access article distributed under the terms of the https://creativecommons.org/licenses/by/4.0/).

Some members of the *Orthoflavivirus* genus, such as the JEV serogroup (e.g., JEV and WNV) (except St. Louis encephalitis virus (SLEV), which lacks the frameshift site [58]) [59,60], *Tamanavirus* genus (Tamana bat virus (TABV), and the highly divergent flavi-like viruses of aquatic animal (fish and crustacean amarilloviruses in Section 3 and Section 4 below) [10,13,21], have two ORFs (ORF1 and ORF2) and produce a “transframe” polyprotein utilizing program3med-1 ribosomal frameshifting (−1 PRF) (Figure 3) [7,13,58]. The eukaryotic mRNA signal for −1 frameshifting comprises two elements. The first element typically consists of a ‘slippery’ heptanucleotide sequence fitting the consensus motif X_XXY_YYZ, where XXX represents any three identical nucleotides; YYY represents AAA or UUU; Z represents A, C, or U; and underscores separate zero-frame codons [58,61]. In the tandem slippage model, the P-site anticodon repairs from XXY to XXX, whereas the A-site anticodon repairs from YYZ to YYY, thus allowing for perfect repairing except at the wobble position. Certain deviations from the canonical XXX of the slippery site are tolerated in the P-site, including UCC in some members of the JEV serogroup [58]. The second element is a 3′ stable RNA secondary structure, such as a pseudoknot or stem-loop, which is separated from the ‘slippery’ heptanucleotide sequence by a ‘spacer’ region typically of 5–9 nt [58,61]. In the JEV serogroup, frameshifting adds a 52 aa transframe C-terminal extension to the NS1 protein, which is not cleaved at the NS1|NS2A cleavage site; thus, frameshifting results in the production of a C-terminally extended version of NS1, known as NS1′ protein [60]. Members of the genera *Orthoflavivirus*, *Hepacivirus*, and *Pegivirus* have two E proteins (E dimers), whereas the genus *Pestivirus* has three (E trimers).

The segmented genomes of the genera *Guaicovirus* and *Jingmenvirus* in the new family *Flaviviridae* encode up to seven structural proteins and two nonstructural proteins (NSP1 and NSP2), which have a high level of similarity to the nonstructural proteins NS5 (RNA-dependent RNA polymerase [RdRp] and methyltransferase [MTase] domains) and NS2B/NS3 (serine protease and helicase), respectively, of all other flavivirid genera (i.e., all genera with non-segmented genomes) [2,35].

**Figure 4:**
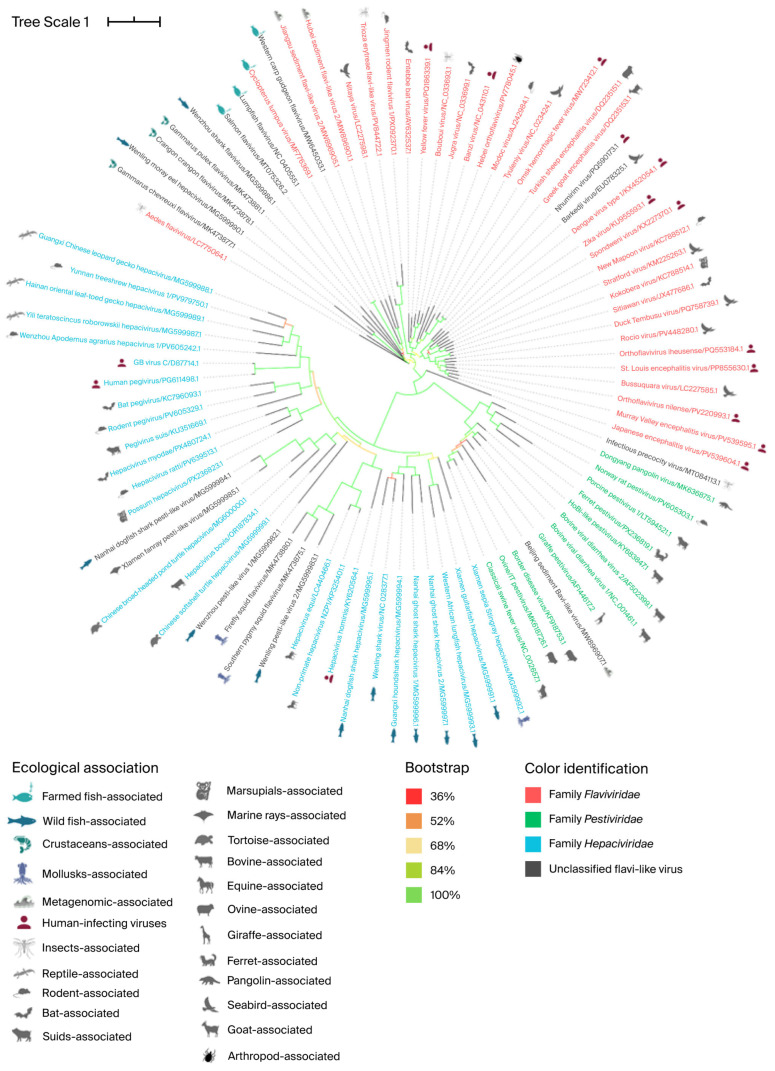
Phylogenetic relationships within the Order *Amarillovirales*. Maximum-likelihood phylogenetic tree of representative members of the order *Amarillovirales* inferred from nucleotide sequence alignments using IQ-TREE2 under the TVMe+R6 model. Branch support was assessed using 1000 ultrafast bootstrap replicates and is indicated by branch color. Taxa are color coded by family, and icons denote host or ecological associations. Branch lengths represent substitutions per site.

A phylogenetic analysis of the polyprotein nucleotide sequences performed in this study is shown in Figure 4. The phylogenetic tree shows that the reported highly divergent flavi-like viruses of aquatic animals (fish and crustacean amarilloviruses in Section 3 and Section 4 below) are found in all three new families: *Flaviviridae*, *Pestiviridae*, and *Hepaciviridae*. However, the grouping is not as clear-cut as with phylogenetic analysis of the RdRp gene [2].

Flavivirids multiply in the cytoplasm and mature into cytoplasmic vesicles derived from the endoplasmic reticulum (ER); assembled virions bud into the lumen of the ER and are secreted through the vesicle transport pathway by the cleavage of prM to M [7]; budding is not seen. Enveloped virions are released through exocytosis [54,62]. In cell cultures examined via electron microscopy, virus replication is commonly accompanied by a characteristic proliferation of intracellular membranes. Infectious precocity virus (IPV) found in farmed giant freshwater prawns, *Macrobrachium rosenbergii*, produced cytoplasmic inclusions in the eyestalk tissue from infected prawns [13].

## 3. Fish Amarilloviruses

It is now generally accepted that fish harbor a large and largely uncharacterized virome [19]. It follows that flaviviruses have been found in a variety of fish species. These viruses, which have been discovered among all four original *Flaviviridae* genera using metagenomics [18,19,48], occur in the three new families, *Flaviviridae*, *Pestiviridae*, and *Hepaciviridae*. Highly divergent amarilloviruses include the pathogenic *Cyclopterus lumpus* virus (CLuV) detected in moribund lumpfish (*Cyclopterus lumpus*) [20] in genus *Tamanavirus*, and Wenzhou shark flavivirus found in both a Pacific spadenose shark (*Scoliodon macrorhynchos*) [48] and a gazami crab (*Portunus trituberculatus*) [10], which group with the new family *Flaviviridae* but are not known to be transmitted by arthropods. The fish viruses that group with the new family Pestiviridae, i.e., fish pesti-like viruses for which full-length polyprotein gene sequences have been published, include Xiàmén fanray pesti-like virus detected in *Platyrhina* sp., Nanhai dogfish shark pesti-like virus in *Squalus brevirostris*, Wenzhou pesti-like virus 1 discovered in *Sphyrna lewini*, and Wēnlǐng pesti-like virus 2 discovered in *Proscyllium habereri* [19,48]. Fish viruses that group with the new family *Hepaciviridae* for which full-length polyprotein gene sequences have been published include Wenling shark virus (WLSV) discovered in the graceful catshark (*Proscyllium habereri*) [18], Wenling moray eel hepacivirus in *Gymnothorax reticularis*, Xiàmén sepia Stingray hepacivirus in *Urolophus aurantiacus*, Western African lungfish hepacivirus in *Protopterus annectens*, Guangxi houndshark hepacivirus in *Mustelus manazo*, and Nanhai dogfish shark hepacivirus in *Squalus brevirostris*, and Nanhai ghost shark hepacivirus 1 and 2 in *Chimaera* sp. [19,48], longfin eel flavivirus and shortfin eel flavivirus 1 found in liver and gill samples of longfin eel (*Anguilla dieffenbachii*) and shortfin eel (*Anguilla australis*), respectively, in New Zealand and most closely related to Wenling moray eel hepacivirus [53]. The best-characterized fish viruses, most of which group with the new family *Flaviviridae*, genus *Orthoflavivirus*, are detailed in the following subsections. In addition, several flavi-like endogenous viral elements (EVEs) (i.e., non-retrovirus EVEs or ‘genomic fossil’) have been identified in various fish species [47,63].

### 3.1. Cyclopterus lumpus Virus (CLuV)

**Figure 5:**
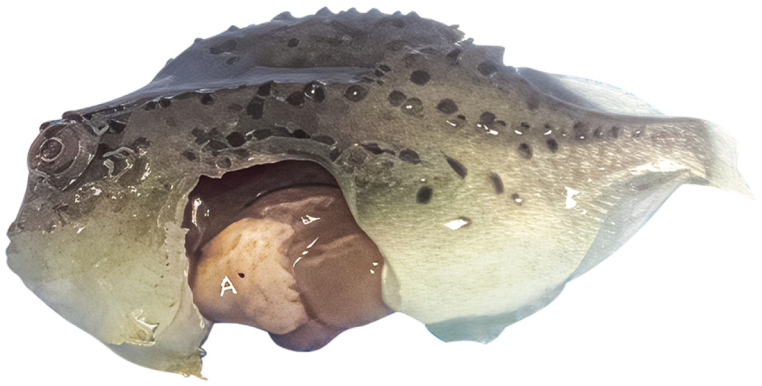
Gross pathology on a lumpfish. Liver is shown to be pale (A), a potential clinical sign of infection with *Cyclopterus lumpus* virus (CLuV). (Reproduced from [33], Figure 1, an open-access article distributed under the terms of the https://creativecommons.org/licenses/by/4.0/).

**Figure 6:**
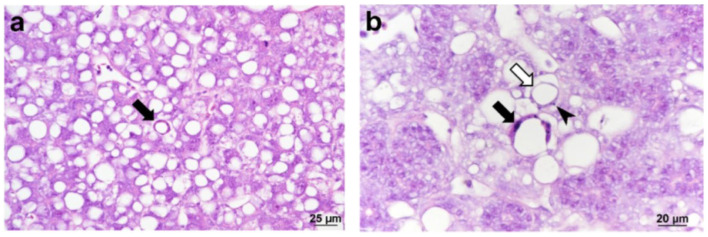
Histological sections of liver showing pathology associated with infection with *Cyclopterus lumpus* virus (CLuV). Hematoxylin and eosin (H&E) stain. (**a**) Hepatocellular apoptosis (arrow), characterized by cellular shrinking and karyorrhexis (nuclear fragmentation). Note the presence of many hepatocytes exhibiting macrovesicular steatosis and displaced cytoplasmic contents. (**b**) Higher magnification of hepatocellular apoptosis clearly showing pyknotic nuclear fragments of karyorrhexis (black arrow). Early onset of apoptosis can be observed (white arrow), where the shrinkage of hepatocytes containing lipid vacuoles results in vacuolation of parenchyma. Note the presence of karyorrhexis (arrowhead), typical of apoptosis. (Reproduced from [33], Figure 2, an open-access article distributed under the terms of the https://creativecommons.org/licenses/by/4.0/).

**Figure 7:**
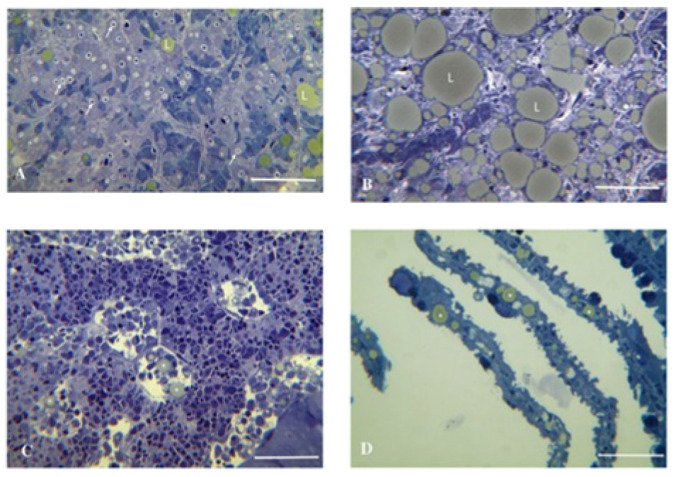
Liver and head kidney pathology associated with CLuV in lumpfish. (**A**) Early stage of disease development showing degeneration of liver cells (arrows) and beginning accumulation of small lipid droplets (L). Bar = 50 μm. (**B**) Terminal stage of the disease, showing massive changes in the liver and accumulation of large lipid (L) inclusions. Bar = 50 μm. (**C**) Section through the head kidney of CLuV-infected lumpfish. Note the presence of cell-associated lipid droplets (asters) in the blood sinuses. Bar = 500 μm. (**D**) Lipid droplets (asters) present in capillaries in gill lamellas. Bar = 500 μm. (Reproduced from [20], Figure 1, an open-access article distributed under the terms of the https://creativecommons.org/licenses/by/4.0/).

*Cyclopterus lumpus* virus (CLuV) is most similar to Tamana bat virus (TABV) [23], species *Tamanavirus parnellis*, which is classified in genus *Tamanavirus* in the new family *Flaviviridae* [2]. CLuV was the first fish flavivirus to be identified. CLuV was first detected in diseased farmed lumpfish (*Cyclopterus lumpus*) in 2015 in Western Norway via next-generation sequencing (NGS) [20]. It is reported that the virus is associated with high mortality (>50%) [20]. Although the virus was present in all tissues tested, pathology was primarily observed in the liver and kidneys. The second detection of CluV was in England using reverse-transcription (RT) quantitative (q) polymerase chain reaction (PCR) (RT-qPCR) developed by Skoge et al. [20] and conventional RT-PCR in September 2021 in consignments of lumpfish imported from Norway, which experienced mortalities of up to 30% [33]. The fish showed typical clinical signs of CLuV-associated disease (such as increased mortalities, lethargy of pre-nursery fish attaching to the side or bottom of tanks, and loss of appetite) [33]. Gross pathology showed pale livers (Figure 5) and histopathology characterized by mild multifocal hepatocellular degeneration (Figure 6) [33] similar to that in the first CluV detection (Figure 7) [20].

Since 2008, lumpfish (*Cyclopterus lumpus*) have been used routinely as “cleaner fish” in marine farmed salmonids as a biological control method for sea lice (*Lepeophtheirus salmonis*) in Norway, UK, Ireland, Iceland, the Faroes, and Canada; the process involves the use of wild-caught cleaner fish directly in the salmon farms or as broodstock for hatchery-raised cleaner fish, and they are often translocated globally [64,65]. The virus in the second detection had 99.44% nucleotide sequence identity compared to the first [33]. However, in the absence of information on the prevalence of CLuV in wild lumpfish populations globally, and with no molecular epidemiological data from Norway [66], it is not known whether the virus had been introduced to the UK farm site via imported juveniles or transmitted to the farmed fish from a local source of the virus. Therefore, the virus may also be endemic in the UK [33].

CLuV cannot be isolated using common fish cell lines such as CHSE-14, ASK [20], BF, EPC, CHSE-214, or E-11 [33], in contrast to salmon flavivirus (SFV), which was isolated on the SSN-1 cell line [21] (see Section 3.5 below). The lack of susceptibility of these cell lines to CluV suggests that its host range is fairly narrow, and there is a need to develop new cell lines from lumpfish to facilitate CLuV cultivation and pathogenesis studies.

### 3.2. Wenzhou Shark Flavivirus

A large-scale metatranscriptomic study discovered 214 vertebrate-associated viruses, including one flavivirus, the Wenzhou shark flavivirus, in the transcriptome of the Pacific spadenose shark (*Scoliodon macrorhynchos*) [48]. This virus was also detected in the transcriptome of healthy gazami crab or Japanese blue crab (*Portunus trituberculatus*), raising the hypotheses of horizontal transmission between the two distantly related hosts in the ocean ecosystem [10] and a correlate with the invertebrate–vertebrate relationship seen in flaviviruses of terrestrial animals [47]. An unconventional flavivirus, most similar to Wenzhou shark flavivirus, *Parastichopus californicus* flavivirus (PcaFV), was detected in Giant Pacific Sea Cucumber (*Apostichopus californicus*; formerly *Parastichopus californicus*) (Holothuroidea; Echinodermata) suffering from wasting disease [17], although the virus was subsequently shown not to cause pathology in its host [67]. It is unknown whether Wenzhou shark flavivirus causes disease in sharks or crabs. However, in sharks infected with the virus, the virus was abundant throughout all tissues tested [10], and other aquatic animal flaviviruses such as CLuV [20,33] and IPV [13] are associated with disease in their natural hosts.

### 3.3. Eastern Red Scorpionfish Flavivirus

A metatranscriptomic study of seemingly healthy fish sold at a fish market in Sydney revealed fragments of a novel aquatic animal flavivirus, Eastern red scorpionfish flavivirus, in the Eastern red scorpionfish (*Scorpaena jacksoniensis*) gill sample [19]. Eastern red scorpionfish flavivirus polyprotein has 54% amino acid identity to Wenzhou shark flavivirus polyprotein [19].

### 3.4. Western Carp Gudgeon Flavivirus

*Flaviviridae* transcripts were detected in the western carp gudgeon (*Hypseleotris klunzingeri*) in the Bogan River as part of the metatranscriptomic viral survey of invasive and native fishes across the Murray–Darling Basin in Australia [22]. These transcripts made up 2% of all vertebrate-associated viruses identified in the samples; the viral sequence exhibited 33–36% NS5 amino acid sequence similarity to other aquatic animal flaviviruses, Tamana bat virus (TABV) [23], species *Tamanavirus parnellis*, which is classified in the genus *Tamanavirus* in the new family *Flaviviridae* [2,21,22].

The viruses identified in Section 3.3 and Section 3.4 were part of metagenomic studies with conflicting observations, one study [19] demonstrating cross-species transmission, with the number of viruses in a fish species related to the fish population density, supporting the view that fish harbor a large and largely uncharacterized virome [19], and the other study [22] demonstrating a lack of virus exchange between native and invasive freshwater fish in the same environment. These metagenomic studies imply that care must be taken in interpreting the significance of new virus discoveries based solely on viral genomic data.

### 3.5. Salmon Flavivirus (SFV)

Salmon flavivirus (SFV) is classified in Lineage Ie in the new family *Flaviviridae* [2]. SFV was isolated from diseased migrating adult Chinook salmon (*Oncorhynchus tshawytscha*) from the Eel River, California, USA, in November 2015 [21]. The affected fish showed signs of lethargy, congregating at the riverbanks, decreased avoidance of humans, and lenticular opacity due to cataract formation, associated with the presence of eye flukes *Diplostomum* sp. trematode metacercariae [21]. Examination of the affected salmon revealed petechial hemorrhages in the optic lobes, cerebellums, and spinal cords that were also observed microscopically [21]. The necropsied fishes’ hearts, livers, spleens, kidneys, intestines, and skeletal muscles exhibited no significant changes. The cytopathic virus was isolated from brain tissue using the striped snakehead (SSN-1) fish cell line, and the complete genome sequence was obtained via next-generation sequencing and rapid amplification of cDNA ends [21]. However, the experimental infection of fingerling rainbow trout (*Oncorhynchus mykiss*) and Chinook salmon via the immersion and intracoelomic injection routes resulted only in limited replication in tissues and no pathology, indicating that SFV is nonpathogenic to Chinook salmon and rainbow trout [21]. Interestingly, ongoing sampling in 2019 revealed the continued presence of the virus in California wild adult salmon, as the virus was isolated from the ovarian fluids of returning females [21].

## 4. Crustacean Amarilloviruses

Flaviviruses have been found in various marine crustacean species. *Crustaflavivirus infeprecoquis* (infectious precocity virus, IPV) was identified via metatranscriptomic sequencing of samples from farmed giant freshwater prawns, *Macrobrachium rosenbergii*, suffering from iron prawn syndrome (IPS) [13]. Three putative flaviviruses were identified from wild-caught malacostracan crustaceans by querying assembled crustacean transcriptomes for flavivirus polyprotein sequences using the tblastn algorithm [10]: *Crangon crangon* flavivirus (CcFV), species *Orthoflavivirus alphei*, genus *Orthoflavivirus* [2], was identified in the brown shrimp (*Crangon crangon*) from midgut samples originating from Weser estuary, Germany [30], *Gammarus chevreuxi* flavivirus (GcFV) was identified in transcriptomes from two publications on *Gammarus chevreuxi* in both embryonic and adult samples originating from the Plym estuary, Plymouth, United Kingdom [50,51] and in *Gammarus pulex* flavivirus (GpFV) identified from a male *Gammarus pulex* wild-caught from the Bourbre River, France [52]. The polyproteins of these viruses were more closely related in terms of amino acid identity to the genus *Orthoflavivirus* [10]. Changjiang-Jingmen-like virus was identified in crayfish [18] and is classified in the genus *Jingmenvirus* in the new family *Flaviviridae* [2].

### Crustaflavivirus infeprecoquis (Infectious Precocity Virus, IPV)

*Crustaflavivirus infeprecoquis* (Infectious precocity virus, IPV) causes iron prawn syndrome (IPS) in farmed giant freshwater prawns *Macrobrachium rosenbergii*, characterized by clinical signs of sexual precocity and stunted growth, which were first observed on farms in Jiangsu Province, China, from 2018 to 2020. IPV was shown to belong to a new genus named *Crustaflavivirus*, denoting a virus genus in the family *Flaviviridae* first identified in crustaceans [13], which corresponds to Lineage Ij in the new family *Flaviviridae* [2].

**Figure 8:**
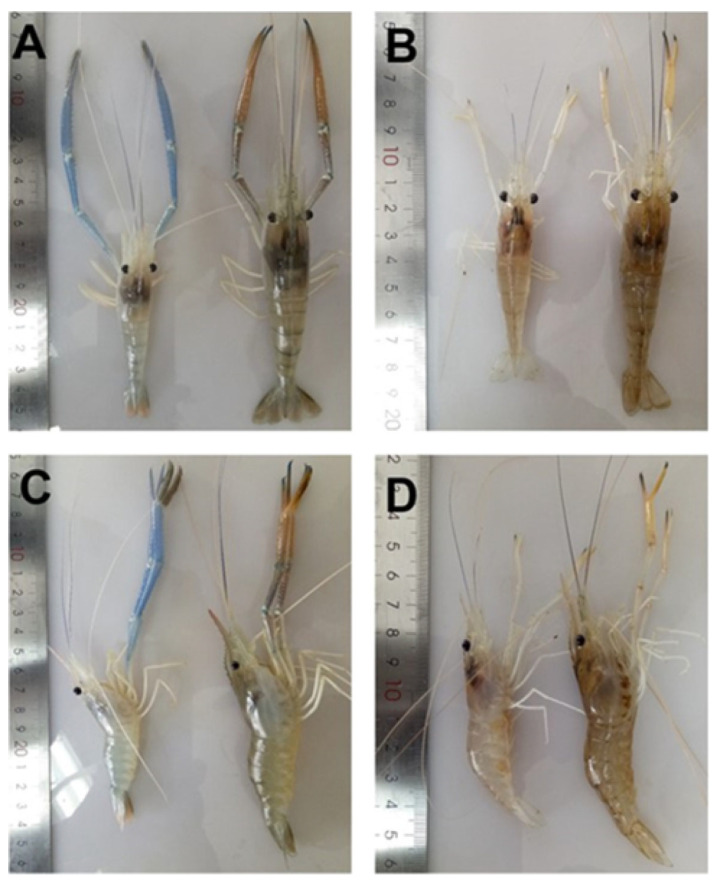
Gross signs of *M. rosenbergii* challenged with IPV preparation. (**A**,**C**, **left**) An infected male *M. rosenbergii*. (**A**,**C**, **right**) A control male. (**B**,**D**, **left**) An infected female *M. rosenbergii*. (**B**,**D**, **right**) A control female. (Reproduced from [13], Figure 2. This is an open-access article distributed under the terms of the https://creativecommons.org/licenses/by/4.0/).

**Figure 9:**
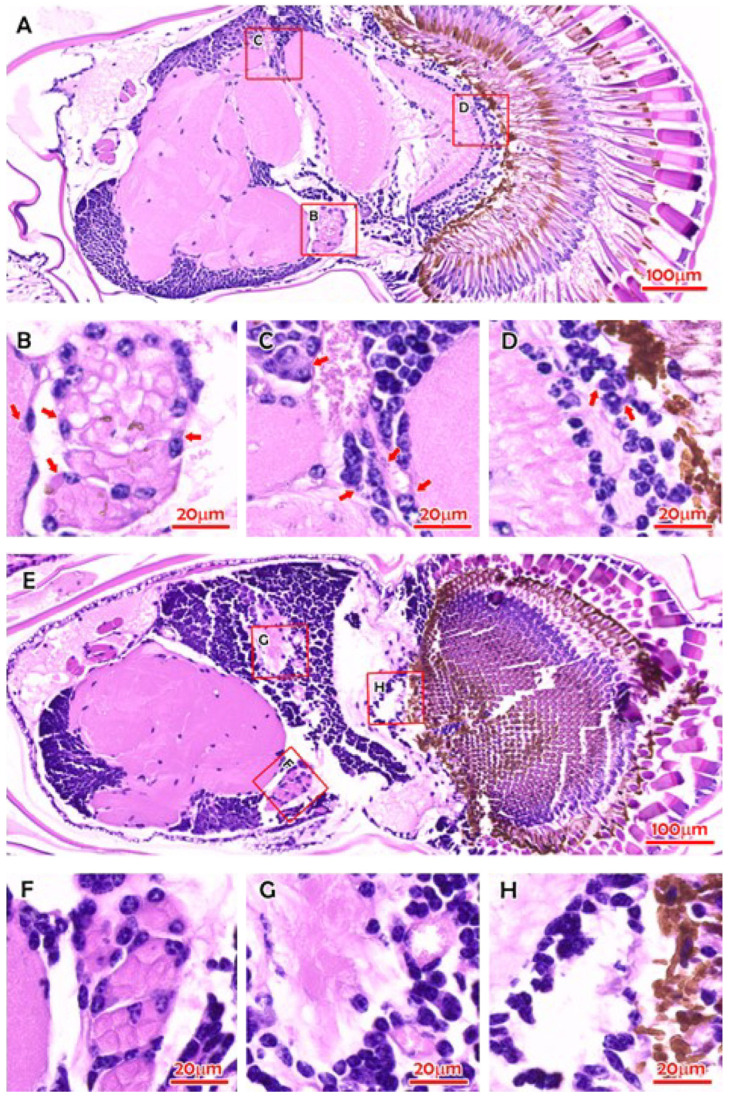
H&E-stained histological sections of tissues of *M. rosenbergii*. (**A**) The overall view of a compound eye of *M. rosenbergii* (0821007) with IPS. (**B**) The magnified onion body with eosinophilic inclusions and reduced membrane layers. (**C**) Cells in the sinus gland area with inclusions. (**D**) Cells in the cortical glia. (**E**) The overall view of a compound eye of *M. rosenbergii* (0821006) without IPS. (**F**) The magnified onion body. (**G**) Cells in the sinus gland area. (**H**) Cells in the cortical glia. Red arrows indicate the eosinophilic inclusion bodies. Bar in panels (**A**,**E**), 100 μm; bar in panels (**B**–**D**,**F**–**H**), 20 μm. (Reproduced from [13], Figure S2. This is an open-access article distributed under the terms of the https://creativecommons.org/licenses/by/4.0/).

Since 2010, IPS has been frequently reported in populations of farmed *M. rosenbergii* in China [13,68,69]. China is the world’s largest *M. rosenbergii*-producing country [70]. IPS is part of the growth retardation disease (GRD) in China, characterized by precocity-associated growth retardation [13,71], which results in substantial production losses in the prawn farming industry [72]. Infected prawns grow to only 5–6 cm, resulting in a 50% loss of yield. The affected female prawns show precocious puberty, i.e., they begin holding eggs when they are up to only 5 cm in body size [71]. The affected males engage in mating behaviors and have two elongated front claws (second pereiopod) when their body length is about 6 cm, while normal mature prawns are 8 to 10 cm long [71]. The condition could be reproduced experimentally by immersion of *M. rosenbergii* post larvae in filtered IPV preparation of the IPS-affected prawns. However, clinical signs did not manifest for at least 22–25 weeks [13]. Figure 8 shows the gross signs of IPS-affected *M. rosenbergii* [13]. Note the elongated blue claws and blue tailfins of the affected prawns; the infected prawns were significantly (*p* < 0.05) smaller than those in the control group after the 22nd week postinfection. Figure 9 shows the histopathology in IPS-affected *M. rosenbergii*, including eosinophilic viral inclusions in multiple tissues [13,69]. In situ hybridization using IPV digoxigenin (DIG)-labeled RNA probes found hybridization signals corresponding with the histopathology in the compound eyes from IPS-affected *M. rosenbergii* [13].

IPS is a persistent, long-term infection characterized by subtle morphological changes that are difficult to detect [73]. It does not cause significant mortality, and clinical signs only occur in the post larvae, making clinical diagnosis extremely difficult as the retardation in growth needs to be differentiated from the normal development and maturation of prawns in the population. Histopathological examination is more reliable as the virus forms eosinophilic cytoplasmic inclusions in the cells of the lamina ganglionaris and fasciculated zone, as well as neurosecretory cells in the bellonci organ and globuli cells in the hemielipsoid body [13].

Several Polymerase chain reaction (PCR) assays for the detection of IPV in samples have been developed [13,74,75,76]. Table 2 lists the PCR primer sequences used in those assays. Dong et al. [13] developed a nested reverse transcription-PCR diagnostic assay specific for IPS-affected prawns that can be used for quarantine inspection for IPV in the transboundary trade of live *M. rosenbergii* and enhanced surveillance of IPV in aquaculture in China and globally. Wang et al. [73] developed a semiquantitative approach for diagnosing IPS based on clinical signs. They established a rapid one-step, highly sensitive, and highly specific reverse-transcription quantitative PCR (TaqMan-RT-qPCR) method to detect and quantify IPV. The detection limit of the TaqMan-RT-qPCR method was as low as 1.00 × 100 copy/reaction. This assay, about 13 and 1300 times more sensitive than the nested RT-PCR assay, revealed the characteristic neurotropism of IPV with the highest loads in eyestalks and the brain [73]. More recently, Chiang et al. [74] used an immunohistochemistry (IHC) assay to localize IPV in hemocytes in the hepatopancreas, gills, and pleopods and in support cells within the lamina ganglionaris as well as in neurosecretory cells within the bellonci organ, sinus glands, and the X organ of infected prawns with or without clinical signs. Chiang et al. [74] then used RT-qPCR with four different primer pairs (Table 2) and the primer pair of Dong et al. [13] and SYBR Green detection to quantitate viral loads in prawns identified as IPV-positive and IPV-negative based on the IHC assay. IHC-positive prawns had viral loads of >10^3.5^ copies/μg compared to <10^3.5^ copies/μg in IHC-negative prawns [53]. Moreover, the highest viral loads were detected in pleopods, which can serve as non-lethal samples for IPS diagnosis [74].

The IPV TaqMan-RT-qPCR assay [73] detected low copies of IPV RNA in red swamp crayfish *Procambarus clarkii* samples [71], suggesting that *P. clarkii* if in the same breeding pond with IPS-affected *M. rosenbergii* may be a reservoir of IPV. Moreover, *P. clarkii* also has clinical signs similar to IPS, such as sexual precocity and stunted growth [65]. However, whether these clinical signs are caused by infection with IPV needs to be further verified [73]. Zhao et al. [75] developed a TaqMan probe RT-qPCR and a nested RT-PCR assay, which were then used to determine the host range of IPV in China. IPV was detected in the crustaceans *M. rosenbergii*, *M. nipponense*, *Procambarus clarkii*, *Litopenaeus vannamei*, *Penaeus monodon*, and *Oratosquilla oratoria* and insect *Anisops kuroiwae* [75]. IPV was not detected in the fish *Misgurnus anguillicaudatus*, *Carassius auratus*, *Oreochromis mossambicus*, *Micropterus salmoides*, or *Pangasius bocourti*; crabs *Eriocheir sinensis* and *Scylla paramamosain*; and malacostraca *Charybdis feriatus* [75]. In *M. rosenbergii*, IPV mainly infected the brain, abdominal nerve, integument, and gill [76]. Regardless, the high sensitivity of the IPV TaqMan-RT-qPCR assay [73,76] means that it is very useful for screening all potential sources of introduction of IPV into *M. rosenbergii* aquaculture [69].

It was recently reported that IPV occurs in two distinct phylogenetic clades, the Southeast Asian clade (represented by isolates 01/SEA/202305 and 02/SEA/202401, GenBank Acc. Nos. PQ786402 and PQ786403, respectively) and the Chinese clade (represented by isolates MR2018, ZJJS2019, ZJHY201110, and JSYZ20170815, GenBank Acc. Nos. MT084113.1, ON382579.1, MT648663.1, and MT648664.1, respectively) [77]. The recent IPV isolate from China, 01/SH/202210 (GenBank Acc. No. PQ786404), exhibited features from both groups, suggesting that it may represent an evolutionary intermediary between the two IPV lineages [77].

## 5. Concluding Remarks

The increased application of viral metagenomics and sequencing activity to commercially important fisheries and aquaculture samples has led to the discovery of novel viruses in aquatic animals, thereby broadening the host range of several viral families of terrestrial vertebrate viruses—in this case, contributing to the reorganization of the family *Flaviviridae* and the incorporation of the highly divergent flavi-like viruses into three new families, *Flaviviridae, Pestiviridae*, and *Hepaciviridae*, in the established order *Amarillovirales*. This review clearly demonstrates that a wide range of amarilloviruses occur in aquatic animals. Some, such as *Cyclopterus lumpus* virus (CLuV) detected in moribund lumpfish (*Cyclopterus lumpus*) and infectious precocity virus (IPV) found in iron prawn syndrome (IPS)-affected farmed giant freshwater prawns (*Macrobrachium rosenbergii*)*,* are important causes of disease in the aquaculture industry and should be considered alongside other flavivirids of human and veterinary medical importance.

As the emergence and spread of serious diseases are the most feared threats to aquaculture, future studies should attempt to develop new aquatic animal cell lines to facilitate virus isolation and propagation of these novel viruses, to allow more research on the pathogenicity of these viruses and their role in the health of aquatic animals. Moreover, these aquatic amarilloviruses may have similarities with those of terrestrial animals that advance our understanding of viral diseases. Additionally, more research is needed to understand the host–virus interactions and the potential occurrence of inter-class viral transmission between the aquatic and terrestrial vertebrates, given that the aquatic amarilloviruses fall basal to those of terrestrial vertebrates. Such studies would place host-jumping, viral disease emergence, and associated zoonotic risk assessments in a broader evolutionary context [78].

## Figures and Tables

**Table 1:** List of highly divergent ‘flavi-like’ viruses (amarilloviruses) found in aquatic animals ^1^.

New Family	New Genus ^2^	Species	Virus Name	GenBank Acc. No	Host	Reference(s)
*Flaviviridae*	*Orthoflavivirus* (Sub-genus *Euflavivirus*)	***Orthoflavivirus dengue***	Dengue virus type 2 (DENV-2)	U87411	Primates and insects	[2]
	*Orthoflavivirus* (Sub-genus *Crangovirus*)	*Orthoflavivirus alphei*	Crangon crangon flavivirus (CcFV)	MK473878	Brown shrimp (*Crangon crangon*)	[2,10,49]
			*Gammarus chevreuxi* flavivirus (GcFV)	QCH00712.1	Gammaridean amphipod (*Gammarus chevreuxi*)	[10,50,51]
			*Gammarus pulex* flavivirus (GpFV)	QCH00716.1	Gammaridean amphipod (*Gammarus pulex*)	[10,31]
			Wenzhou shark flavivirus (WZSFV)	AVM87250.1	Pacific spadenose shark (*Scoliodon macrorhynchos*); gazami crab or Japanese blue crab (*Portunus trituberculatus*),	[10,48]
			Eastern red scorpionfish flavivirus (ERsfFV) *	MH716818	Ray-finned fish eastern red scorpionfish (*Scorpaena jacksoniensis*)	[19]
	*Tamanavirus*	***Tamanavirus parnellis***	Tamana bat virus (TABV)	AF285080	Parnell’s mustached bat (*Pteronotus parnellii*)	[2]
	*Tamanavirus*		*Cyclopterus lumpus* virus (CLuV)	MF776369	Lumpfish (*Cyclopterus lumpus*)	[20,33]
			Lumpfish flavivirus (LuFV) *	NC_040555	Lumpfish (*Cyclopterus lumpus*)	[20]
			Western carp gudgeon flavivirus (WCgFV) *	MW645033	Western carp gudgeon (*Hypseleotris klunzingeri*)	[22]
	Lineage Ie		Salmon flavivirus (SFV)	MT075326.2	Chinook salmon (*Oncorhynchus tshawytscha*)	[2,21]
	Lineage Ij		Infectious precocity virus (IPV)	MT084113	Giant freshwater prawns (*Macrobrachium rosenbergii*)	[2,13]
			*Gammarus pulex* flavivirus (GpFV)	MK473881	Gammaridean amphipod (*Gammarus pulex*)	[10,52]
	*Jingmenvirus*	***Jingmenvirus rhipicephali***	Jīngmén tick virus (JMTV)	KJ001579—KJ001582	Ticks and mammals	[2]
	*Jingmenvirus*		Changjiang Jingmen-like virus	APG76081	Crayfish	[18]
*Pestiviridae*	*Orthopestivirus*	***Orthopestivirus bovis***	bovine viral diarrhea virus 1 (BVDV1)	M96751	Cattle	[2]
			Wenzhou pesti-like virus 1 (WZPLV-1) *	MG599982	Scalloped hammerhead (*Sphyrna lewini*)	[19,48]
			Wēnlǐng pesti-like virus 2 (WLPLV-2) *	MG599983	Graceful catshark (*Proscyllium habereri*)	[19,48]
			Nanhai dogfish pesti-like virus (NDfPLV) *	MG599984	Japanese shortnose spurdog (*Squalus brevirostris*)	[19,48]
			Xiàmén fanfray pesti-like virus (XFfPLV) *	MG599985	Fanrays (*Platyrhina* sp.)	[19,48]
*Hepaciviridae*	*Orthohepacivirus*	***Orthohepacivirus hominis***	hepatitis C virus (HCV) genotype 1a	AF009606	Humans	[2]
			Wenling shark virus (WLSV)	NC_028377	Graceful catshark (*Proscyllium habereri*)	[18]
			Xiàmén guitarfish hepacivirus (XgHCV) *	MG599991	Ringstreaked guitarfish (*Rhinobatos hynnicephalus*)	[19,48]
			Xiàmén sepia Stingray hepacivirus (XsSHCV) *	MG599992	Sepia stingray (*Urolophus aurantiacus*)	[19,48]
			Western African lungfish hepacivirus (WAlHCV)	MG599993	West African lungfish (*Protopterus annectens*)	[19,48]
			Guangxi houndshark hepacivirus (GhHCV)	MG599998	Starspotted smooth-hound (*Mustelus manazo*)	[19,48]
			Nanhai dogfish shark hepacivirus (NdshHCV) *	MG599995	Japanese shortnose spurdog (*Squalus brevirostris*)	[19,48]
			Nanhai ghost shark hepacivirus 1 (NgshHV 1)	MG599996	Ghost sharks (*Chimaera* sp.)	[19,48]
			Nanhai ghost shark hepacivirus 2 (NgshHV 2)	MG599997	Ghost sharks (*Chimaera* sp.)	[19,48]
	Lineage IIIt		Wenling moray eel hepacivirus (WmeHCV)	MG599990	Moray eel (*Gymnothorax reticularis*)	[2,19]
			Longfin eel flavivirus (LeFV) *	OR863209	Longfin eel (*Anguilla dieffenbachii*)	[53]
			Shortfin eel flavivirus 1 (SeFV1) *	OR863218 and OR863219	Shortfin eel (*Anguilla australis*)	[53]

^1^ New flavivirid taxonomy of Order *Amarillovirales* [2] with aquatic animal virus members and established type species of the genera shown in **bold** for context; genera may contain additional members and potential species. ^2^ Unnamed genera designated Lineages in clades I and III corresponding to families *Flaviviridae* and *Hepaciviridae*, respectively. * not a recognized abbreviation.

**Table 2:** List of PCR primer sequences used for detection of Infectious precocity virus (IPV) in samples.

PCR Assay	Primer Name	Primer Sequence (5′→3′)	Amplicon Length (bp)	Reference
Nested RT-PCR	IPV_F1	5′-GCACACTCCCAACACGTTTC-3′	1038	[13]
	IPV_R1	5′-CGCGCGTAATCTCTACACCT-3′		
	IPV_F2	5′-TCCCTAGGCAGGGGATACTG-3′	395	
	IPV_R2	5′-AGCTATCCGTGGTGTGGAAC-3′		
TaqMan-RT-qPCR	IPV-F	5′-AGGAGAGGGTTTTGGCTTG-3′	139	[73]
	IPV-R	5′-CTGGATTGGAAGGGAACTCTG-3′		
	IPV-P	5′-[6FAM]-CCGCGACACTTACAACTGCCC TT-[TAMRA]-3′		
SYBR Green RT-qPCR	6872F	5′-AAGAATTCGGAGTCTATGTTGACGGC-3′	525	[74]
	7396R	5′-AACTCGAGCACTTTCCTACCCG-3′		
	6871F	5′-GGAGTCTATGTTGACGGCTCTATCT-3′	256	
	7127R	5′-GTTGGTGAACCTATGATCCTCTTA-3′		
	7396F	5′-GCTGAGAAAGCGGGTAGGAAAGT-3′	205	
	7601R	5′-ACCACGACAACATCATAGGTAAAGG-3′		
	7828F	5′-GTGTTCTGTCCTAGTGCAGTTGG-3′	220	
	8048R	5′-CGCGCCATGAAGCCATAATAAC-3′		
Nested RT-PCR	IPV-F1	5′-GCCTCCACATCATTGGCTTCG-3′	754	[75]
	IPV-R1	5′-TCGGGTGTCATCAACAAACTCATA-3′		
	IPV-F2	5′-ACATCATTGGCTTCGTAT-3	395	
	IPV-R2	5′-ACAGAGCAGGAGATTGGA-3′		
TaqMan-RT-qPCR	IPVq-F	5′-GAAGATGTCATCGTCCCAGAGTT-3′		
	IPVq-R	5′-GGAATGCCCCCTCCGTAT-3′		
	probe	5′-CCCCAAGGTTTTATTG-3′		

## Data Availability

The original contributions presented in the study are included in the article; further inquiries can be directed to the corresponding author.
